# Over-expression of secreted proteins from mammalian cell lines

**DOI:** 10.1002/pro.2439

**Published:** 2014-03-11

**Authors:** Annamarie C Dalton, William A Barton

**Affiliations:** Department of Biochemistry and Molecular Biology, Virginia Commonwealth UniversityRichmond, Virginia, 23298

**Keywords:** secreted protein, receptor, protein expression, mammalian cell culture

## Abstract

Secreted mammalian proteins require the development of robust protein over-expression systems for crystallographic and biophysical studies of protein function. Due to complex disulfide bonds and distinct glycosylation patterns preventing folding and expression in prokaryotic expression hosts, many secreted proteins necessitate production in more complex eukaryotic expression systems. Here, we elaborate on the methods used to obtain high yields of purified secreted proteins from transiently or stably transfected mammalian cell lines. Among the issues discussed are the selection of appropriate expression vectors, choice of signal sequences for protein secretion, availability of fusion tags for enhancing protein stability and purification, choice of cell line, and the large-scale growth of cells in a variety of formats.

## Introduction

In the post-genomic era, the need for highly purified recombinant protein for biophysical and biochemical studies is greater than ever. In this regard, the laboratory workhorse, *Escherichia coli*, has been essential for the simple low-cost expression of the majority of proteins investigated to date. However, a number of biomedically relevant proteins fail to express and fold properly in prokaryotic expression hosts. For example, mammalian secreted proteins and membrane bound receptors often contain obligate post-translational modifications including disulfide bonds and unique glycosylation patterns that are required for proper folding and/or biological activity, preventing their expression in prokaryotes. Alternatively, a number of powerful eukaryotic expression systems are available for expression of challenging proteins including those that contain unique post-translational modifications. The most common over-expression platforms currently include yeast (e.g., *Pichia pastoris* and *Saccharomyces cerevisiae*), baculovirus expression vector systems (*Autographa californica* multiple nuclear polyhedrosis virus and insect cell hosts *Spodoptera frugiperda* or *Trichoplusia ni*), and mammalian cell systems (including a variety of transformed cell lines such as CHO and HEK293). It should be noted that each expression platform has its own merits and disadvantages, and one may be more suitable for a particular protein over others.

## Yeast Over-Expression System

Yeast over-expression systems have a number of advantages. In many cases, yeast systems have provided exceptionally high yields of secreted recombinant protein while also being relatively inexpensive and time efficient.^1^–^5^ Microbiological culture media can be an order of magnitude less expensive than insect or mammalian cell culture media, and yeast can be cultured to high density in reusable glass Erlenmeyer shake flasks or cultured at higher density in oxygen sparged fermenters. Furthermore, the time commitment required for development of an expression cell line is relatively low and as cell doubling is rapid, expression experiments are often relatively straight-forward and quick. Unfortunately, yeast express a number of proteases which can lead to significant, yet often protein dependent, protein degradation.^3^,^4^ A number of protease deficient cell lines are now readily available if needed. Nonetheless, both *Pichia* and *Saccharomyces* construct high-mannose type N-glycosylations on proteins and are incapable of producing the more complex patterns often seen in mammalian proteins.^6^ Depending upon the protein of interest and intended use of the protein (e.g., structural studies vs. functional assays), this may or may not be an important issue. Thus, although yeast represents a robust over-expression system for many secreted proteins, their divergence from native mammalian post-translation modifications and variability in levels of expression renders them problematic for routine protein production.

## Insect Cell (Baculovirus) Expression System

The baculovirus expression vector system is the principal method for production of challenging cytosolic proteins that are unable to be effectively synthesized in prokaryotic hosts.^7^,^8^ In contrast, their use in expression of secreted mammalian proteins is far more limited for a variety of reasons. First, insect cell culture media is as costly as mammalian cell culture. Although both insect cells and mammalian cells can be routinely cultured in serum containing classical media, chemically defined media, or serum-free media, the overall cost of these formulations is nearly identical for the two cell types. Intuitively, the potential for near native-like glycosylation patterns is significantly higher in mammalian cells (e.g., insect cells are unable to produce sialylated complex glycans) as is often the overall protein yield.^7^,^9^ Second, the dependence on viral transduction presents a number of fundamental issues that are amplified when considering over-expression of secreted proteins. Although viral production has been significantly streamlined over the past few years, particularly in development of the recombinant viral backbone using bacterial homologous recombination, viral production and amplification still represent time-consuming steps prior to protein expression trials.^7^,^8^ In addition, once the baculovirus is established and amplified to produce a viral stock prior to a large-scale expression experiment, it must be tittered and an appropriate multiplicity of infection must be established to determine optimal expression. Construction, amplification, tittering, and optimization are lengthy steps and often require a month or more. Moreover, viral stocks have limited shelf life, are unable to be frozen without significant loss in titer, and are often consumed on production of a limited amount of protein before the virus need once again be amplified, tittered, and tested. Finally, and perhaps most importantly, baculovirus infected insect cells often yield lower amounts of secreted proteins than mammalian cell lines.^7^ Upon infection, the baculovirus genome (which includes the transgene expression cassette) is significantly amplified while host synthesis ceases. Despite having to compete with viral genes, the expression cassette, frequently also under control of a strong viral promoter (i.e., the polyhedron promoter) is itself transcribed and translated at exceptionally high levels. Complex secreted proteins require a number of host factors including glycosylation machinery, chaperones for folding, and disulfide isomerases to name just a few. Under these conditions, the insect cell host machinery is often remarkably overwhelmed, thus secreted proteins fail to substantially fold and are instead frequently found trapped inside the cell in large aggregates.^10^,^11^ Interestingly, co-expression of the ER-resident chaperone BiP/Grp78 with a protein disulfide isomerase can enhance secreted expression in insect cells.^12^–^15^ Indeed, trafficking through the secretory pathway remains the primary bottleneck for all eukaryotic expression systems. Under conditions of extreme over-expression as observed in the baculovirus expression system, greater intracellular expression can result in lower yields of folded and secreted protein.

## Mammalian Cell Over-Expression System

Mammalian cell culture is the prevailing method for biopharmaceutical protein production and its use is growing in popularity among academic laboratories.^10^,^16^–^19^ Intuitively, mammalian cell hosts are more likely than lower eukaryotic cell hosts to express, properly fold, and yield native-like post-translational modifications of secreted mammalian proteins. Glycosylation patterns from over-expressed secreted proteins produced in mammalian cells are most often consistent with that observed *in vivo* and are relatively homogeneous in nature, albeit with minor differences between different species of cell hosts.^16^,^18^,^20^ Furthermore, stable mammalian cell lines, in particular, represent a reusable resource that can be stored under cryogenic conditions for long periods of time (potentially indefinitely), retrieved, and cultured to provide a consistent and reliable level of protein expression. However, despite the widespread use of mammalian cells for secreted protein over-expression in the pharmaceutical industry, academic labs have been relatively slow to adopt mammalian expression systems in part because of the relatively scarce detailed protocols that are directly applicable to academic production of protein on the milligram to hundreds of milligram scale.

In this review, we discuss the many choices and possibilities encountered when planning protein over-expression experiments in mammalian cells including transient versus stable expression, the selection of expression vector elements, signal sequences, cell lines, media formulations, and methods for large-scale growth.

## Stable Chromosomal Integration Versus Episomal or Transient Expression

The type of over-expression system used is primarily dictated by the ultimate downstream application of the protein under investigation, and even then, the investigators requirements may change over time. Preliminary expression experiments for structural analysis, for example, commonly explore a number of protein variants including homologues from different species, truncations at the amino or carboxyl-terminus, or specific mutations known to influence structure or function. To analyze a large number of protein variants quickly, transient expression experiments are most advantageous to obtain small amounts of protein for initial biochemical characterization (light scattering, e.g., to monitor protein aggregation). Alternatively, once an appropriate variant(s) has been identified, large quantities of homogeneous protein is required for setting crystallization trials, which may include the addition of binding partners and ligands. Under these conditions, the investment required to generate a stable clonal cell line would be warranted, and perhaps, even be ideal.

Stable mammalian cell expression can be driven either when the transgene (expression cassette) is replicated extra-chromosomally using viral proteins and *cis*-acting elements, or when integrated into the host genome. Stable expression of the transgene is generally desirable for large-scale production and control over protein quality and homogeneity. Alternatively, large-scale transient transfection experiments have grown in popularity over the past few years, primarily for the capacity to analyze a number of protein constructs rapidly.^21^–^24^ However, several issues may limit the general utility of this technique. For example, transient expression experiments require a sizeable quantity of consumable reagents including transfection reagent and plasmid DNA, both of which can be financially prohibitive. Additionally, protein yield is dependent on the transfection efficiency, a factor that can vary considerably from one experiment or individual to the next. This issue can be particularly problematic for large-scale experiments since expression is not directly scalable as it is for stable cell lines. Perhaps most importantly, protein transiently produced from older cells may not be qualitatively equivalent to protein produced from cells of earlier passage due to the transformed nature of the cell lines used thus potentially increasing protein heterogeneity and decreasing protein quality.^10^,^16^,^18^,^20^ Finally, it is often argued that transient expression is less time-consuming than generating a stable cell line. Single transient expression experiments undeniably require a smaller time commitment than does establishing a stable clonal cell line. However, in the event several transient transfections must be repeated, the combined time commitment and cost of continual transfection will offset the initial time commitment required for stable cell line production.

As mentioned above, stable transgene expression can be maintained using one of two distinct strategies. Episomal vectors based on viral elements promote autonomous replication and retention in the nucleus (e.g., Epstein–Barr virus [EBV] or the BK virus [(BKV]). Episomal vectors rely on a *cis*-acting element (viral origin of replication) and *trans*-acting virally encoded protein.^25^,^26^ Transgene amplification can be extensive, but in general varies dramatically with copy numbers ranging from 20 to 150 for BKV or approximately 5 to 30 for EBV. This variability is one factor that may make them a less desirable alternative to stable chromosomal integration.^25^

**Figure 1:**
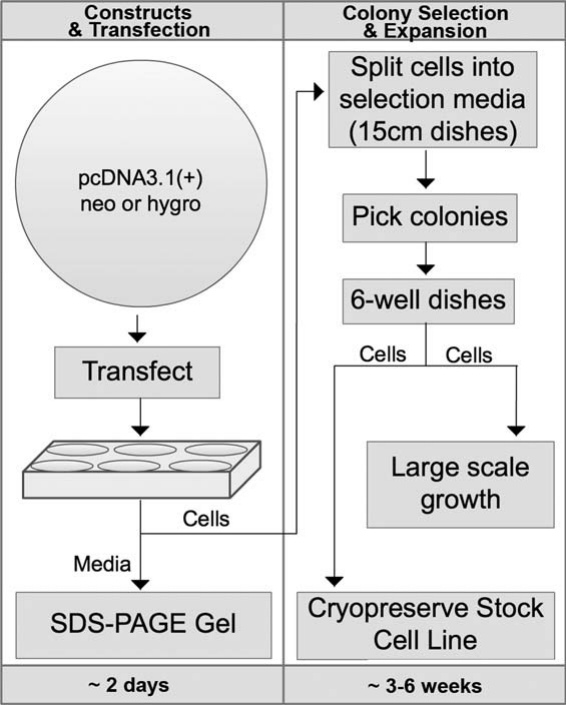
Schematic representation of steps involved in over-expression of secreted proteins from stable mammalian cell lines. Expression constructs are initially evaluated by transient expression and SDS-PAGE in six-well plates. Approximately 48–72 h post transfection, cells are split directly into media containing selection reagent, for example. Cultures are maintained in selection media for approximately 8–14 days before visible colonies appear and can be cloned by traditional ring cloning techniques. Individual clones are expanded into six-well plates and tested for expression. Acceptable clones are expanded in larger dishes and an aliquot is preserved and cryogenically frozen. At this point, cell stocks are ready for large-scale growth.

In contrast to episomal expression, stable chromosomal integration and expression relies only on the presence of a suitable selection marker for isolating and screening clonal cell lines (Fig. 1). A wide array of selection systems have been developed including the commonly used methotrexate/DHFR and glutamine synthetase protein expression systems as well as the more conventional use of selectable drug markers.^27^,^28^ In these systems, stable expression is maintained by physically coupling the transgene expression cassette to a dominant selection marker. Positive cells are selected and cloned using either limiting cell dilution or *via* physical isolation with cloning cylinders. A noted drawback of this method is that the process of chromosomal integration is generally thought to be a random event, which in some cases results in gene silencing. Indeed, integration “position effects” are profound and result from differential chromatin modifications to loci DNA. Thus, its anticipated that levels of protein expression will vary from clonal lines produced in this manner necessitating clonal screening. Nonetheless, in general, stable clonal cell lines are an attractive expression platform that provides a variety of advantages, though most notably, a reliable and consistent yield and quality of recombinant protein.

## Expression Vector Elements

Vectors used for stable transfection studies include a number of essential elements that promote optimal transcription, processing, RNA export, and translation. Here, we briefly discuss issues relating to the choice of transcription regulatory sequences (i.e., promoter), as well as the selection of signal sequence for protein secretion, use of fusion tags, choice of drug selection marker, and reporters of gene expression.

Mammalian expression vectors share in common a robust promoter/enhancer element for high levels of transcription. Most widespread are the strong viral promoters from cytomegalovirus (CMV) and SV40. Many commercial vectors also utilize the human EF-1α promoter although expression from viral promoters, at least in some cases, is considerably higher.^29^ In addition to the promoter element, several *cis*-acting elements both within and outside the coding region are recognized to greatly effect cellular expression. For example, the Kozak sequence, which includes the initiating methionine, and in higher vertebrates is the consensus sequence gccRccAUGG, plays a role in translation initiation.^30^,^31^ Alterations within this region decrease the efficiency of translation initiation and can have profound physiological effects.^32^ Alternatively, sequences within the 3′ non-coding region can also influence gene expression. For example, the viral post-transcriptional regulatory sequence from the woodchuck hepatitis virus (WPRE) was found to enhance viral titer and transgene expression when placed downstream of an open-reading frame in the sense orientation.^33^ Interestingly, recent studies have shown that the WPR element functions in nuclear export in a CRM1-dependent manner and protein expression levels can increase by approximately twofold when placed in the appropriate orientation.^29^,^34^,^35^ Similarly, the presence of introns has been known for greater than 40 years to enhance gene expression with increases in expression occasionally being quite dramatic (up to 400-fold), although in most circumstances the increase is more modest (approximately twofold).^29^,^36^,^37^ Finally, a poly-adenylation signal derived from SV40 or bovine growth hormone is commonly found downstream of the open-reading frame and is essential for efficient transcriptional termination and 3′ processing. Elements affecting poly-adenylation include splicing elements and structured regions near the signal sequence both of which can slow termination, processing, and promote nuclear accumulation of mRNAs.^29^,^38^

### Signal sequence selection

Secretory peptides include residues at the amino-terminus of a protein that destine the nascent peptide for translocation into the ER. Following co-translational insertion of the growing peptide chain into the ER lumen, a signal peptidase cleaves the signal peptide from the protein. Perhaps not surprisingly, the proper selection of a signal peptide can have dramatic consequences on protein over-expression with some investigators reporting up to fourfold enhanced levels of expression.^38^,^39^ Intuitively, the best choice for signal sequence may be the proteins native signal peptide unless truncations from the amino terminus are to be explored. In either case, testing a small panel of commonly utilized signal sequences may be desirable. A handful of efficient and well-described signal sequences include interleukin-2, CD5, the Immunoglobulin Kappa light chain, trypsinogen, serum albumin, and prolactin, although there are many others that have proved beneficial as well.^10^,^38^,^39^ While some signal peptides appear general in their ability to promote protein secretion of a variety of proteins, others are more protein specific.^39^ Thus, empirical trials may be useful if expression levels are low.

### Fusion tags

**Figure 2:**
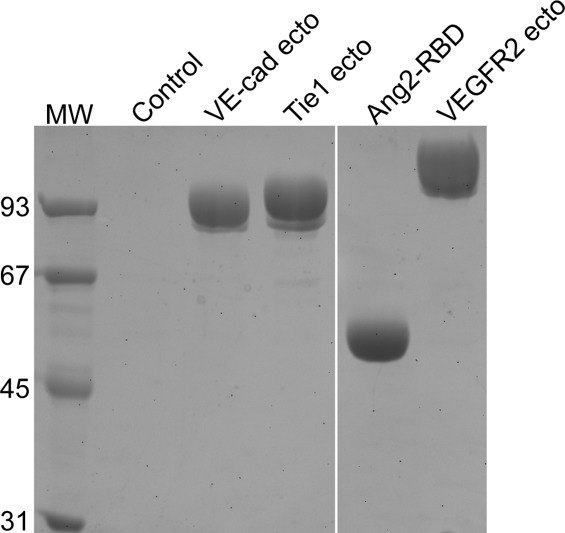
SDS-PAGE analysis of various Fc-tagged proteins transiently expressed from 293 cells. One milliliter of media was harvested 48 h post-transfection and Fc-tagged fusion protein was immunoprecipitated with Protein-A Sepharose and electrophoresed on a 10% SDS-PAGE gel. Molecular weight markers are shown on the left.

Fusion tags are frequently used to optimize protein folding and stability but can also provide a convenient means for single-step purification with high yield. Several fusion proteins are used extensively for cytoplasmic protein expression experiments, yet significantly fewer are available for secreted protein expression. Indeed, the only commonly used fusion partners for secreted proteins include human serum albumin and the crystallizable fragment, or constant domain of IgG, Fc.^40^,^41^ The Fc tag is typically fused to the carboxyl-terminus of proteins of interest and can have a dramatic effect on overall expression (Fig. 2).^40^ The histidine tag is also frequently used to simplify purification of recombinant protein from the media, although no evidence suggests it has the ability to enhance protein expression.

### Drug selection marker

For stable or episomal protein expression, an efficient drug selection marker is required. The most common selection reagents for mammalian cells are neomycin/G418, hygromycin, blastocidin S, zeocin, and puromycin. Neomycin and hygromycin resistance, coded for by an aminoglycoside phosphotransferase and hygromycin B phosphotransferase, respectively, are the most frequently cited drug markers and are present in a number of commercially available vectors.^42^ However, in our hands, selection with neomycin yields a high degree of clones that despite displaying drug resistance, fail to express the transgene. Cell death in the presence of neomycin is also substantially slower than many of the other reagents. The same is not true for hygromycin, which efficiently kills cells within a few days of drug selection and results in a high percentage of clones expressing the transgene of interest (approaching 100%). Thus, for our studies, we primarily utilize hygromycin (resistance coded for by the hygromycin B phosphotransferase), although puromycin (resistance coded for by the puromycin *N*-acetyltransferase) is also frequently used with good results.^43^

### Reporters of expression

Reporters of expression can provide a facile and non-invasive means to monitor protein expression *in vivo*. For membrane proteins, for example, the fluorescence signal from GFP when introduced as a carboxyl-terminal fusion is useful for following the extent of protein expression and potential for aggregation.^24^ While not directly applicable to secreted proteins, several labs have demonstrated that co-expression and intracellular fluorescence of GFP from downstream IRES elements can serve as a decent predictor of gene expression in transient expression experiments.^44^ Furthermore, cap-independent translation from IRES elements does not appear to significantly reduce cap-dependent translation from the upstream expression cassette.^45^ However, transcriptional activity and mRNA abundance poorly correlate with protein expression and due to the inherent differences in the bottlenecks imposed on expression of cytoplasmic versus secreted proteins, intracellular markers such as GFP serve as poor indicators of secreted protein levels and for stable expression experiments, GFP is no more beneficial than the original selection marker used to select stable clones.^46^

## Cell Line Selection

A number of transformed mammalian cell lines have been explored for protein over-expression studies. By far the most extensively utilized cell line is the dihydrofolate reductase deficient Chinese hamster ovary cell line CHO-DG44.^47^ Other popular lines also include the Adenovirus 5 transformed human embryonic kidney cell line, HEK293, the SV40 transformed African green monkey CV-1 line, COS-1, and the non-Ig secreting sub-clone of NS1 cells, NS0. Specific cell lines each have their own potential benefits as well as possible drawbacks. For example, CHO cells are tremendously popular for biopharmaceutical production in conjunction with methotrexate induced DHFR gene amplification.^28^,^48^ Yields of antibodies and recombinant proteins from CHO cells are typically higher than all other cell lines.^10^ However, DHFR/methotrexate mediated gene amplification, selection, and screening of high-secreting clones is prohibitively laborious and time-consuming for academic labs. Similarly, NS0 cells express low levels of glutamine synthetase, and thus their viability is often coupled to glutamine synthesis through the glutamine synthetase selection marker for cell line selection.^16^,^27^ Derived from B cells, the NS0 line is primarily utilized for antibody expression, though its routine use in protein production is growing. In contrast to all other cell lines mentioned here, COS-1 cells are generally limited to transient expression experiments.^25^,^26^ Introduction of vectors containing the SV40 origin of replication into COS-1 cells, which express the SV40 large tumor antigen (T-Ag), leads to efficient and rapid vector and transgene amplification. A significant increase in gene expression is realized due to increased copy number, and high levels of expression can be maintained for several days before cell viability all but ceases.^49^

Academic labs have embraced the use of HEK293 cells in protein over-expression studies for a number of reasons. HEK293 cells grow robustly and are easily transfected by a number of reagents with efficiencies routinely exceeding 50% using linear polyethylenimine, or approaching 100% with commercial lipid formulations.^50^–^52^ Furthermore, HEK293s readily adapt from growth as an adherent monolayer to suspension and can be cultured in a wide variety of classical and serum-free media.^53^ In addition, several HEK293 cell line variants have been produced including those deficient in glycosidases (Gtn1-) and those with enhanced adherence to culture surfaces (GripTite), which may be beneficial for biophysical studies including crystallographic structure determination.^54^ HEK293T cells, a variant that stably express SV40T-Antigen, are also readily available and used frequently for transient expression studies, though they are much less frequently used for stable gene expression.

## Large-Scale Mammalian Cell Culture

The methods used for large-scale cell growth primarily depend on the properties of the expression host. For example, HEK293 cells are a particularly attractive expression host because they readily adapt from adherent culture to suspension, thus a variety of options are available for their culture. For example, HEK293 cells grown as an adherent monolayer in disposable plastic tissue culture vessels are ideal for single cell cloning experiments, while growth in suspension in shake flasks is beneficial for pilot expression experiments and can be invaluable for large-scale protein production trials. Adherent cells are also adaptable to a wide variety of culture conditions in large volumes using either plastic or glass roller bottles, on hollow fiber reactors, on the surface of microcarriers, or polyester fiber disks as seen in packed bed bioreactors.

**Figure 3:**
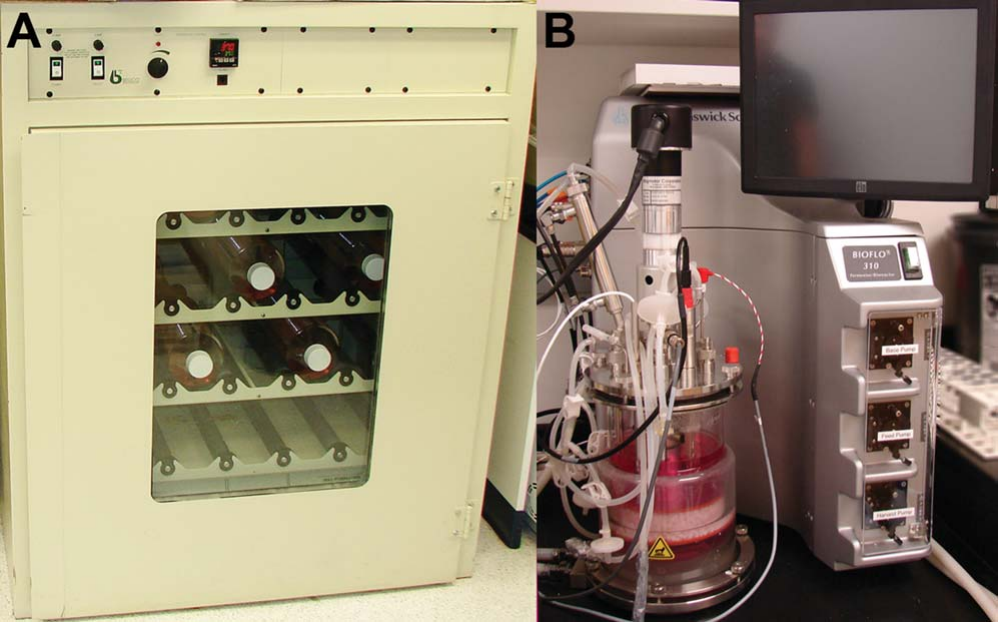
Large-scale growth of adherent 293H cell lines. (A) A mini 37°C roller incubator can hold up to 40 expanded surface pleated roller bottles at a time (equivalent to 10 L of media). (B) For larger volumes, 293H cells can be efficiently grown in a fiber cell packed-bed bioreactor similar to the BioFlo 310 for weeks to months at a time using either fed-batch or perfusion culture techniques.

For production of milligram quantities of secreted protein from 1 to 10 L of culture media, we favor growing adherent monolayers in disposable plastic roller bottles [Fig. 3(A)]. We have found that for routine protein production experiments, roller bottles allow a convenient and readily scalable method to produce between 1 and 100 mg of protein for crystallization trials. Pleated or expanded surface roller bottle are readily available from a number of manufacturers, which significantly increase the growth surface area and hence amount of secreted protein that can be harvested from each bottle. A small roller incubator can hold approximately 40 bottles, or the equivalent of 10 L of culture media, while larger incubators can handle as many as 100 bottles, or the equivalent of 25 L of media. With the potential for each bottle to contain a cell line expressing a different protein or protein variant, numerous proteins can be produced simultaneously for efficient crystallization trials, for example. Thus for academic labs, roller bottle culture may represent the highest throughput, lowest cost, and highest yield for protein expression experiments.

For expression studies, bioreactors are unrivaled for optimal mammalian cell growth. Typical bioreactors are sophisticated equipment capable of monitoring and optimizing numerous environmental variables simultaneously for improved cell growth [Fig. 3(B)]. Packed bed bioreactors using Fibra-Cel**®** polyester fiber disks, for example, have the capacity to grow adherent cells to exceptionally high cell density (estimated at approximately 1 × 10^8^ cells/mL), far greater than that which can be obtained in suspension.^55^,^56^ Protein yields from bioreactors are typically several fold higher than that from cells grown in roller bottle culture or suspension, and individual cultures can be maintained for weeks to months, which may be particularly attractive for proteins that express poorly or are needed in extremely large quantities.^55^–^57^ Nonetheless, bioreactors represent a substantially higher initial and continuing investment. Moreover, bioreactors are by necessity low throughput and hence only truly effective in academic settings for cell lines with relatively low overall yields. They also represent a substantial time investment since cleaning, setup, and breakdown often require more than a few days. In general, bioreactors are less than ideal for most academic labs that require the ability to express many different proteins or protein variants simultaneously, and instead are best for continual culture of cell lines producing low yields of secreted protein.

## Media Formulations and Optimization

A number of manufacturers produce both classical and serum free media for most commonly used expression cell lines. For example, the media of choice for routine adherent culture of HEK293 cells is Dulbecco's modified Eagle's media containing >4.5 g/L glucose, sodium pyruvate, and fetal bovine serum (FBS) at 5–10%.^57^ Alternatively, for suspension cultures, Joklik's modified minimal essential media containing 5–10% horse, bovine, or FBS is recommended.^58^ The selection of media is most often one of cost, cell type cultured, method of cell culture (adherent vs. suspension), and presence or absence of affinity tag for purification purposes. Serum-free media is now becoming common for routine suspension culture. For cell lines prone to aggregation in suspension, formulations of serum-free media can minimize cell clumping relative to classical media containing serum. One issue to note, in lieu of serum, chemically defined or serum-free media often contains animal or plant protein hydrosylates in addition to purified factors such as insulin and transferrin.^53^,^59^,^60^ Historically, most cell lines have been cultured in media supplemented with bovine serum, or FBS at levels of 5–10%. Although 10% FBS provides the highest cellular viability and best growth rates, 5% FBS is occasionally acceptable for routine purposes. Alternatively, depending on cell type, bovine serum may be a cost-effective supplement for routine culture of adherent and suspension cells. Interestingly, HEK293 cells may be particularly attractive because early studies demonstrated that 10% horse serum can effectively promote cell growth in suspension culture.^58^ Nevertheless, despite these alternatives FBS is almost exclusively used during over-expression experiments since high cellular viability and growth rate are prerequisites for obtaining high protein yields.

To further enhance protein expression during expression experiments, expression media can be supplemented with small molecules such as sodium butyrate or valproic acid.^61^–^63^ Sodium butyrate has been shown to increase protein yield by as much as 50% presumably through its ability to block histone deacetylation. Allen and colleagues recently screened a variety of small molecules for their ability to influence gene expression and identified a number of attractive compounds, including hexanohydroxamic acid.^61^ Although the majority of compounds appear to modify host histone acetylation or methylation patterns, they do not all seem to effect gene expression at the transcriptional level. Although some evidence suggests that many small molecule effectors are beneficial for protein expression studies, they also appear relatively cell line specific, which may necessitate empirical trials for your cell line of interest.

Finally, seleno-methionine incorporation is an attractive means to prepare a heavy atom derivative for phase determination by the multiwavelength anomalous diffraction method in X-ray crystallography. Cell culture media specifically lacking methionine is readily available and has recently been used to incorporate the unnatural amino acid seleno-methionine into secreted proteins produced in mammalian cells at labeling efficiencies approaching 90%.^64^ Although other cell lines may be directly adapted for this purpose, the above-mentioned protocol was developed specifically using HEK293 cells.

## Conclusion

Mammalian cells provide an attractive alternative to prokaryotic hosts for difficult to express proteins including secreted proteins. Purification of large quantities of secreted mammalian proteins is readily attainable, though the methods required differ significantly from those used for membrane and cytosolic proteins. Unfortunately, many of the tools that have been developed for optimizing expression and tracking of cell bound molecules are not helpful when screening for secreted proteins. Here, we have discussed some of the techniques that make it possible to maximize secreted protein quantity in a facile manner, including roller bottle or bioreactor culture and secretion specific fusion tags. Additionally, with careful design of the expression cassette and minimal optimization of parameters including signal sequence, cell type, and media, robust over-expression can be readily obtained for most secreted proteins.
